# Pediatric Risk Mapping From Co‐Exposure to Extreme Temperatures and Air Pollutants

**DOI:** 10.1029/2025GH001743

**Published:** 2026-05-30

**Authors:** Jagadeesh Puvvula, Jonathan J. Szeto, Jabeen Taiba, Jennifer Ko, Jesse E. Bell, Wei‐Ting Hwang, Rebecca A. Simmons, Marilyn Howarth, Sameed Ahmed M. Khatana, Kyle Jackson, Aimin Chen

**Affiliations:** ^1^ Department of Biostatistics, Epidemiology and Informatics Perelman School of Medicine University of Pennsylvania Philadelphia PA USA; ^2^ The Leonard Davis Institute of Health Economics University of Pennsylvania Philadelphia PA USA; ^3^ Perelman School of Medicine University of Pennsylvania Philadelphia PA USA; ^4^ Department of Environmental, Agricultural, and Occupational Health College of Public Health University of Nebraska Medical Center Omaha NE USA; ^5^ School of Natural Resources University of Nebraska‐Lincoln Lincoln NE USA; ^6^ Daugherty Water for Food Global Institute University of Nebraska Lincoln NE USA; ^7^ Center for Research on Reproduction and Women's Health University of Pennsylvania Philadelphia PA USA; ^8^ Department of Pediatrics Perelman School of Medicine University of Pennsylvania Philadelphia PA USA; ^9^ Division of Neonatology Children's Hospital of Philadelphia Philadelphia PA USA; ^10^ Division of Cardiovascular Medicine Perelman School of Medicine University of Pennsylvania Philadelphia PA USA; ^11^ Penn Cardiovascular Outcomes, Quality, and Evaluative Research Center Perelman School of Medicine University of Pennsylvania Philadelphia PA USA; ^12^ Division of Transplant Surgery Hospital of the University of Pennsylvania Philadelphia PA USA

**Keywords:** extreme temperature, air pollution, risk mapping, children

## Abstract

Exposure to extreme temperatures and fine particulate matter is hazardous to human health, especially among children. With growing evidence on the impact of these environmental hazards on child health—including increased risks of respiratory illnesses, heat‐related illnesses, and developmental challenges—there is a substantial need to identify high‐risk areas for potential adverse pediatric health outcomes. In this study, we developed pediatric vulnerability indices at the census tract scale across the contiguous United States (CONUS) using five dimensionality reduction methods, including unsupervised machine learning and deep learning approaches. We integrated these indices with data on extreme temperature (2012–2024), PM_2.5_, and black carbon exposures from 2010 to 2020, enabling spatial analysis of environmental hazards and pediatric vulnerability. Among the five methods tested, principal component analysis (PCA) was selected for its balanced representation of 12 vulnerability factors, which explained 23% of the variance in the data. We observed that co‐exposure to extreme temperature and air pollutant exposures were highest in the West, Southwest, and certain parts of the South and Northeast. Approximately 36% of the CONUS showed statistically significant hotspots of co‐exposures to higher temperatures and air pollutants, concentrated in the West, Northwest, and Southwest. High‐risk areas for pediatric vulnerability and co‐exposure to environmental hazards were identified in both urban and rural communities, including indigenous lands and agricultural regions. These findings can aid policymakers and public health officials as a preliminary resource in developing heat action plans and allocating cooling centers to protect children living in the most affected communities.

## Plain Language Summary

Children are particularly vulnerable to health issues associated with high temperatures and air pollution. In this study, we sought to identify the areas in the United States that pose the greatest risk to children from both extreme heat and air pollution. We created an index to measure children's vulnerability across neighborhoods, using data on family income, access to healthcare, and other social factors. We also looked at data on high temperatures and air pollution from 2012 to 2024. The results of this work indicate that children living in the West, Southwest, and certain parts of the South and Northeast are at a higher risk of adverse health outcomes due to extreme temperatures and air pollution. Approximately one‐third of the country's area experienced both high temperatures and high air pollution simultaneously. These areas included both cities and rural places, as well as indigenous lands and farming communities. Our results help decision‐makers and health officials as a preliminary resource for allocating resources, such as cooling centers, and for developing plans to protect children during periods of extreme heat and high air pollution.

## Key Points

- We developed a census tract scale pediatric vulnerability index by comparing five dimensionality reduction methods
- Co‐exposure to extreme heat and high air pollutants was primarily concentrated in the Western, Southwestern, and Southern CONUS regions
- High‐risk hotspots were observed across the West, South, Southeast, and parts of the Northeast and Midwest

## 1 Introduction

Exposure to ambient particulate matter with a diameter of less than 2.5 μm (PM_2.5_) and extreme heat is associated with a range of adverse health outcomes in children (Hellden et al., 2021; Liu et al., 2024; Schapiro et al., 2024). In the United States, daily PM_2.5_ concentrations are moderately correlated (*r* = 0.56) with daily maximum ambient temperatures, suggesting a potential compounding effect on human health (Zhang et al., 2017). Concurrent exposure to extreme temperatures and air pollutants can have synergistic effects, exacerbating the risk of adverse human health outcomes through oxidative stress, airway inflammation, and systemic physiological stress responses (Coker et al., 2025; Rahman et al., 2022; Rai et al., 2023). Children are particularly vulnerable to environmental hazards due to their developing organs and physiological characteristics, including higher respiratory rates, immature metabolic pathways, and incomplete development of detoxification mechanisms (Ahdoot et al., 2024).

This vulnerability is further amplified for children living in socioeconomically disadvantaged neighborhoods, where exposures are disproportionately higher and health impacts more pronounced due to the co‐occurrence of other socioeconomic factors associated with adverse health (Assari & Zare, 2024; Heard‐Garris et al., 2021; Jee & Patel, 2025; Landrigan et al., 2010; Morello‐Frosch et al., 2011). Data from the National Survey of Children's Health indicate that one in eight children face multiple hardships, such as food, housing, and medical care insecurity, with these vulnerabilities disproportionately affecting racial and ethnic minority populations (National Survey of Children's Health, 2023). The formerly United States Global Change Research Program (USGCRP) emphasized that population characteristics—including race, poverty, education, housing quality, and access to healthcare—play a critical role in shaping both adaptive capacity and resilience to environmental hazards (Gamble et al., 2016). These complex interactions between environmental hazards and population vulnerabilities highlight the importance of integrated risk frameworks for heat–health risk assessment, such as that employed by the Intergovernmental Panel on Climate Change (IPCC), which explicitly considers the interaction between hazard, exposure (overlapping areas with hazard and vulnerability), and vulnerability in quantifying environmental risks (Reisinger et al., 2020).

In recognition of the geographic variability in human health risks due to the interaction between environmental hazards and population vulnerabilities, several vulnerability assessment tools have been developed to identify communities at elevated environmental health risk—including the Child Opportunity Index 3.0 (COI 3.0), Environmental Justice Screening and Mapping Tool (EJSCREEN 2.3), Climate and Economic Justice Screening Tool (CEJST 1.0), EPA Environmental Quality Index (EPA‐EQI), USGCRP Climate Mapping for Resilience and Adaptation (CMRA), and NHHIS Extreme Heat Vulnerability Mapping (Council on Environmental Quality, n.d.; Federal Emergency Management Agency, 2023; National Integrated Heat Health Information System, n.d.; C. Noelke et al., 2020; Pradyumna & Sankam, 2022; U.S. Environmental Protection Agency, 2020; U.S. Global Change Research Program, n.d.). While these tools include sociodemographic and environmental factors and serve important functions in broad environmental justice health community planning, each presents distinct limitations when applied to pediatric co‐exposure risk: most operate at coarse spatial resolution of environmental hazards that mask within‐neighborhood microenvironmental heterogeneity; with the exception of COI 3.0, they were designed for adult or general populations rather than pediatric‐specific vulnerabilities; they treat heat and air pollution as separate additive components rather than modeling synergistic co‐exposure effects; and they employ static temperature thresholds that introduce geographic bias by not accounting for regional weather and adaptation patterns. These limitations collectively constrain the ability of existing tools to identify precise neighborhoods requiring more targeted pediatric interventions.

We performed a PubMed query as of 13 August 2025, and identified 66 peer‐reviewed articles (Table S1 in Supporting Information S1). Among these, we identified eight relevant original research articles. Additionally, we manually identified seven additional articles related to the research topic. Among the 15 original articles, a majority (10 of 15) focused exclusively on extreme temperatures, two on air pollutant exposures, and two on both heat and air pollution (Table S2 in Supporting Information S1). Among studies focused on heat vulnerability, most were conducted in a US state or city, except for Reid et al., which covered the entire CONUS. However, Reid et al. focused on land cover as a potential predictor of heat‐related risk and developed a heat vulnerability index at the census tract scale (Reid et al., 2009). Other studies using air temperature were also limited in their geographic extent and relied on a static temperature threshold to define extreme heat, potentially undermining the geographic variability in temperature (Aubrecht & Ozceylan, 2013; Christenson et al., 2017; Eisenman et al., 2016). Studies focused on air pollution vulnerability have been confined to specific regions, such as New York City and Wisconsin (Christenson et al., 2017; Kannoth et al., 2025). Additionally, Anderson et al. developed an overall environmental index at the census tract scale, combining environmental hazards, vulnerability, and baseline health metrics to identify potential hotspots (Anderson et al., 2025).

Overall, we identified gaps in geographic scope, co‐exposure to environmental hazards, methodological heterogeneity for dimensional reduction, and a focus on the pediatric population. To address these gaps, we aim to: (a) develop and compare a census‐tract‐scale pediatric vulnerability index using multiple dimensionality reduction methods; (b) explore spatio‐temporal patterns of extreme heat and air pollutants (PM_2.5_, black carbon), and identify potential hotspots; and (c) identify neighborhoods with high pediatric vulnerability and high exposures using the IPCC risk framework.

## 2 Materials and Methods

### 2.1 Data

#### 2.1.1 Ambient Temperature Anomalies

This study defined daily maximum temperature anomalies as the “frequency of days above the monthly historical normal.” Since the maximum temperature was reported as one of the strongest indicators of human health risks in the US, we used the daily maximum temperature metric in this study (Davis et al., 2016).

##### 2.1.1.1 Historic Climate Normals

Monthly historical (1901–2000) Climate Gridded Data set (NClimGrid) at 1/24° latitude/longitude (∼4 km (km)) spatial resolution for the CONUS was accessed from NOAA's National Center for Environmental Information (NCEI) database (Vose et al., 2014). This monthly data set provides a nearly homogeneous and spatially complete temperature time series across the CONUS. These observations provide a historical context of temperature patterns. In this study, we used the monthly normals of maximum temperature (°F) during the meteorological summer months (June, July, and August) to identify anomalies over the study period (Figure S1 in Supporting Information S1).

##### 2.1.1.2 Daily Maximum Temperature Anomalies (2012–2024)

The daily maximum temperature for the CONUS at ∼4‐km resolution was obtained from the Global Historical Climatology Network‐daily (GHCNd) database Version 1.0 (Durre, Arguez, et al., 2022; Durre, Squires, et al., 2022). These observations are generated by applying thin‐plate smoothing splines to the observations from the GHCNd that passed quality checks (Menne et al., 2012). We calculated the frequency of days above the US Historic Climate Normals during the meteorological summer months of the study period and transformed the anomalies into a tertile scale (Low: 0–33rd percentile, Moderate: 34–67th percentile, and High: 68–100th percentile) to provide an interpretable exposure classification while maintaining balanced group sizes across geographic areas.

#### 2.1.2 Ambient Air Pollutant Anomalies (2010–2020)

We identified air pollutant anomalies using ground‐level daily mean PM_2.5_ and black carbon estimates at a 1‐km resolution, modeled using a spatiotemporally weighted deep forest framework (Wei, Wang, & Li, 2023; Wei, Wang, Li, et al., 2023). Input parameters of the air pollutant model includes, remote sensing observations (aerosol optical depth from remote sensing observations [MODIS, MAIAC MERRA2, MERRA2‐ PM_2.5_/black carbon components]), Copernicus Atmosphere Monitoring Service PM_2.5_/black carbon emissions, meteorologic parameters, Normalized Difference Vegetation Index (NDVI), elevation, population, and spatial/temporal weight terms, to yield daily observations. Modeled estimates demonstrated correlations with measured observations, with R‐squared values of 0.78 (PM_2.5_‐spatial = 0.72; PM_2.5_‐temporal = 0.74) and 0.68 (black carbon‐spatial = 0.68; black carbon‐temporal = 0.78). For PM_2.5,_ anomalies were defined as days with concentrations greater than 35 μg/m^3^ and greater than 1 μg/m^3^ for black carbon. We transformed air pollutant anomalies into a tertile scale, similar to the scale used for ambient temperature anomalies.

#### 2.1.3 Pediatric Vulnerability

We identified population vulnerability metrics from the National Climate Assessment reports (Gamble et al., 2016; Hayden et al., 2023; West et al., 2023). Among these vulnerability metrics, we selected the 12 variables relevant to the pediatric population and available through the US Census Bureau. These variables fall into four themes: race, financial barriers, access to care, and others (Table S3 in Supporting Information S1). We accessed 2021 American Community Survey Data (ACS 5‐Year estimates) through the US Census API (Walker et al., 2021).

Variables such as the proportion of children from racial/ethnic minority groups, those without access to insurance, those with a language barrier, and those living with single parents were estimated as the percentage of children (≤19 years) per census tract. Additionally, vehicle access, federal assistance, income, and poverty were calculated as a percentage of the household scale per census tract. Furthermore, the estimated variables for the overall population scale represent households without access to a vehicle, foreign‐born individuals, and those living below the federal poverty level. All vulnerability metrics were converted to a percentage scale, taking values from 0 to 100 per census tract (*n* = 84,414).

#### 2.1.4 Rural‐Urban Classification of the CONUS

We obtained the 2010 revised Rural‐Urban Commuting Area (RUCA) Codes from the United States Department of Agriculture (USDA, 2019). We then filtered the 48 contiguous States and the District of Columbia (D.C.) (72,392 tracts) and performed a join with the 2010 administrative boundaries shapefile at the census tract scale accessed through the US Census Bureau web API (Walker, 2016). In this study, we used the primary‐level RUCA codes, which range from 1 to 10, to describe a census tract. Following the USDA‐RUCA code structure, we aggregated levels 1–3 as metropolitan, 4–6 as micropolitan, 7–9 as small towns, and 10 as rural areas. This data set also includes 131 census tracts that are not coded to any of the above categories.

### 2.2 Developing Pediatric Vulnerability Index

We constructed latent variables representing the population vulnerability characteristics using five dimensionality reduction methods. Four are unsupervised machine learning‐based, and one is a deep learning algorithm. Using the 12 pediatric vulnerability factors scaled as percentages per census tract, we preprocessed the variables by applying a log_10_ transformation and center‐scaled them to account for potential skewed distributions.

We first performed a principal component analysis (PCA) to yield eigenvalues per component, loading values, and principal component scores per census tract (Krzanowski, 1987). Similarly, we conducted an exploratory factor analysis (EFA) to yield eigenvalues, factor loadings, minimum sampling adequacy, and factor scores per census tract (Haig, 2005). Using bootstrap sampling (*n* = 5,000), we calculated 95% confidence intervals for the percent variance explained using PCA and EFA. Then, using the t‐distributed Stochastic Neighbor Embedding (t‐SNE; model tuning parameters identified through sensitivity testing: perplexity = 40, theta = 0.8, and dims = 3) to yield a stress value of 2.19, we obtained the stress value (model fit metric) and three latent variables per census tract (Hinton, 2003). Furthermore, the Uniform Manifold Approximation and Projection (UMAP) method was applied to yield two latent variables per census tract (McInnes et al., 2018). Finally, an autoencoder algorithm (4 layers: input, 2 dense layers, output layer) was applied, using mean squared error as the loss function and the *Adam* optimizer, to yield two latent variables via an encoder and a dense layer (Rumelhart et al., 1986).

These five dimensionality reduction methods were applied independently to directly compare model performance via metrics such as explained variance (PCA), factor reliability (EFA), stress (t‐SNE), topological preservation (UMAP), or reconstruction error (autoencoder), and to choose the one that aligns with the data structure and index objectives. This rigorous selection process enabled us to choose a method that optimally balances the efficacy of dimensionality reduction and the interpretability of socioeconomic indicators. Specifically, PCA efficiently maximizes variance via orthogonal components while reducing noise, but assumes linearity with less interpretable loadings; EFA (oblimin) uncovers correlated interpretable factors despite subjective retention and sample sensitivity; t‐SNE reveals local clusters with global distortions; UMAP scalable balances local/global structure though parameter‐dependent; and autoencoders flexibly capture nonlinear features, with potential for overfitting without regularization.

Upon applying the dimensionality reduction algorithms to the pediatric vulnerability variables, we obtained 10 latent variables (2 per method). We then compared the strength of the correlation (Spearman) between the latent and the original variables to select a latent variable that was highly correlated with the original variables for further analysis.

### 2.3 Identification of Hazard Trends and Potential Hotspots

We calculated the annual frequency of temperature and air pollutant anomalies for each pixel to explore potential trends over the study period. Temporal trends at each pixel were estimated using linear regression, with year as the predictor and the annual frequency of anomalies as the outcome variable. The estimates for temporal trends were interpreted as an increase every year associated with the frequency of anomalies.

Additionally, we homogenized the extreme temperature raster at 4 km resolution and the fine particulate matter (PM_2.5_ and black carbon) at 1 km resolution to yield a 1 km spatial raster format using the Lanczos resampling method (Hijmans et al., 2022). Using the homogenized hazard data, we identified potential hotspots for high temperatures and fine particulate matter at a 1 km grid scale (*n* = 8,119,702 grids) using the Getis‐Ord Gi* method (Bivand et al., 2011; Ord & Getis, 1995). Weights of the nearest neighbors were defined using the K‐nearest neighbors method, assuming 20 spatial units (Bivand et al., 2011). Furthermore, as a sensitivity analysis, we conducted hotspot analysis assuming 15 and 25 neighbors to identify potential spatial clusters.

### 2.4 Risk (Hazard by Vulnerability) Mapping

In this study, we defined high risk following the IPCC risk framework (Figure S2 in Supporting Information S1) as the areas that overlapped high hazard (areas with high heat and air pollutant levels) and high vulnerability (pediatric population vulnerability characteristics) (Reisinger et al., 2020). Exposures in this study refer to the neighborhoods with overlapping hazards and vulnerability. We merged the hazard layer at 1 km with the vulnerability layer at the census tract scale using a spatial join to harmonize the coordinate systems. This procedure enabled us to utilize the high‐resolution temperature hazard layer instead of aggregating it to administrative boundaries, such as census tracts. We then transformed the hazard co‐exposure variable from 27 levels to 3 levels. Hazard level “high” includes 2 or more hazards in the high category, “moderate” includes a hazard in the high category, and low includes hazards in the low or moderate categories. High‐risk areas were defined as locations with high hazard (elevated temperatures and air pollution levels) that overlap with high vulnerability (populations with characteristics associated with increased susceptibility).

## 3 Results

### 3.1 Pediatric Vulnerability Index

**Figure 1:**
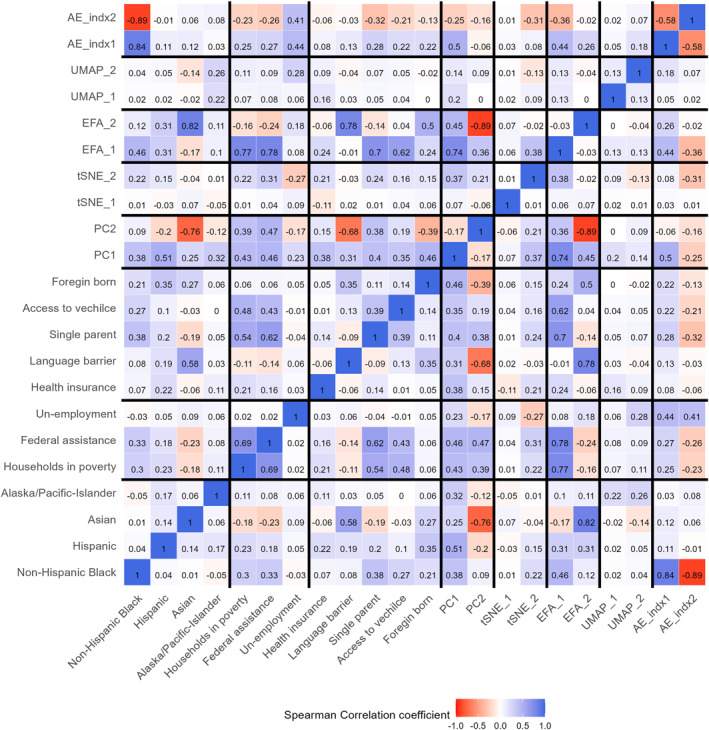
Spearman correlation between social determinant variables and pediatric indices. Pediatric indices are the latent variables generated using the five dimensionality reduction methods. PCA‐principal component analysis, EFA‐exploratory factor analysis, t‐SNE‐t‐distributed Stochastic Neighbor Embedding, UMAP‐Uniform Manifold Approximation and Projection, AE‐autoencoder algorithm. This figure includes two latent variables generated from using five dimensionality reduction methods.

Among the five dimensionality reduction methods, we observed a strong correlation between the latent variables created using the PCA and EFA methods, indicating the best‐represented population vulnerability characteristics (Figure 1). The PCA latent variable explains 22.6% (95% CI: 22.3–22.9) of the variance in the vulnerability data, and the EFA latent variable explained 16.5% (95%CI: 16.1–16.9). However, the two differ in their correlations with the 12 individual factors (Table 1). The latent variable derived from the PCA method showed a consistently modest correlation across all 12 factors (Figure 1; Figure S3 in Supporting Information S1). Meanwhile, the EFA‐based latent variable demonstrated strong representation of variables for children living in households below the federal poverty level and those with single parents. Therefore, we used the PCA‐based latent variable in subsequent analyses of pediatric vulnerability.

**Table 1:** Characteristics of Pediatric Vulnerability Index

Category	Variable	Pediatric index loading values	
		PCA	EFA
Race/Ethnicity	African American	0.23	0.15
	Hispanic	0.31	0.08
	Asian	0.14	−0.07
	Alaska/Pacific‐Islander	0.15	−0.01
Access to Care/Transportation	Health insurance	0.24	0.06
	Access to vehicle	0.33	0.59
Financial vulnerability	Federal assistance (SNAP/Food stamps)	0.36	0.45
	Households in poverty	0.44	0.71
	Un‐employment	0.12	0.04
Other	Language barrier	0.20	0.05
	Foreign born	0.26	0.12
	Single parent	0.44	0.60

*Note*. PCA‐Principal component analysis; EFA‐Exploratory factor analysis; Proportion of variance explained by that first latent variables: PCA = 23% & by EFA = 17%.

### 3.2 Extreme Temperature

**Figure 2:**
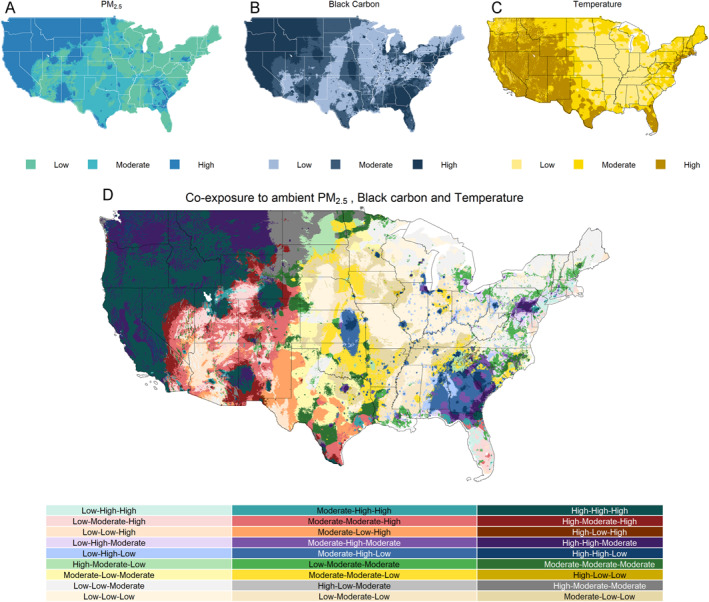
Cumulative exposure to ambient temperature, PM_2.5_ and black carbon in the CONUS. Panel (a) and (b) represent PM_2.5_ and black carbon anomalies over 2010–2020. Panel (c) represents maximum temperature anomalies during the meterological summer months (JJA) over 2012–2024. Panel (d) represents the co‐exposure to the three environmental hazards. This figure includes exposures on a tertile scale to indicate environmental hazards as low, moderate, and high. The category “low” for the PM_2.5_ exposures includes null values. This figure legend includes a combination of three metrics, the first component represents “PM_2.5_,” the second represents “black carbon,” and the third represents “temperature.”

Over the study period (2012–2024), we observed a majority of the areas in the West (CA, NV), the Northwest (OR, ID), the Southwest (UT, CO, AZ, NM), the Northern Rockies and plains (WY), South (TX), Southeast (FL), and the Northeast (NJ) had a higher number of extreme temperature anomalies (Figure 2). Among these, the Southwestern states and Nevada had higher cumulative exposure to temperature throughout the state. During the study period, we observed that certain states in the Southwest (UT, AZ), South (TX, LA, MS), and Southeast (AL, GA, FL) had relatively higher warming trends compared to the other CONUS states (Figure S2 in Supporting Information S1). In contrast, we observed a cooling trend in certain rural areas of the Ohio Valley (MO, IL, IN, KY).

### 3.3 Air Pollutant Anomalies

Over the last decade (2010–2020), we observed high PM_2.5_ concentrations in the West (CA, NV), the Northwest (OR, WA, ID). Northern Rockies and plains (MT, WY, ND, SD), and some areas of Arizona, New Mexico, Colorado, Tennessee, Georgia, South Carolina, and Pennsylvania. We observed null trends or weak improving trends (in MT, IA, PA, GA) of PM_2.5_ concentrations across the CONUS, except for a weak increasing trend of PM_2.5_ concentrations in the West (CA, NV), the Northwest (WA, OR), and certain regions in Colorado, Wyoming (Figures S3 and S4 in Supporting Information S1). In contrast, we observed a decline in PM_2.5_ concentrations in Idaho and in some areas of California, Oregon, Washington, Utah, and New Mexico.

The cumulative exposure to black carbon showed a distinct trend compared to PM_2.5_ concentrations. In addition to the West (CA, NV), the Northwest (OR, WA, ID), where we observed high PM_2.5_ concentrations, we observed higher concentrations of black carbon in the South (spanning across the Flint Hills area across KS, OK), the Southeast (AL, GA, SC), and the Northeast (PA, CT). However, we observed a declining trend in black carbon concentrations across the CONUS, except in certain areas of California, Nevada, Arizona, and New Mexico, which showed an increase in black carbon concentrations (Figure S5 in Supporting Information S1).

### 3.4 Co‐Exposure to Extreme Temperature and Air Pollutants

**Figure 3:**
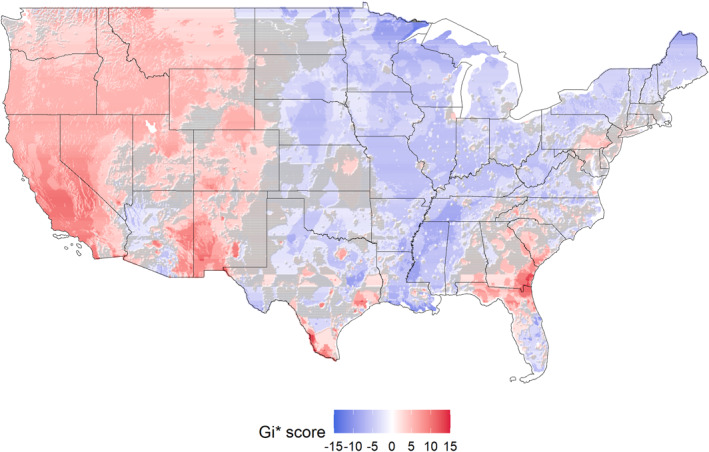
Localized hotspots of co‐exposure to air pollutants and extreme temperature. This figure includes Gi* score generated using the Getis‐Ord Gi* hotspot analysis. The Gi* values range between −15 and 15, where a positive Gi* values indicate higher concentrations of air pollutants and extreme temperature and negative values indicate lower concentrations. Gray color represents statistically non‐significant trends.

We observed 36% of the CONUS with statistically significant hotspots (higher co‐exposure to extreme temperatures and air pollutants), about 40% with cold‐spots (lower exposure), and about 24% with null results. Most hotspots are located in the West (CA, NV), Northwest (OR, WA, ID), driven by high exposure to temperature and air pollutants, and the Southwest (part of AZ, NM), driven by high exposure to air pollutants/moderate to high temperatures (Figure 3). Additionally, we observed certain regions in Colorado, Wyoming, and Montana with high exposures to air pollutants/moderate to high temperatures. Furthermore, we observed certain regions of the coastal Southwest and the eastern region of Pennsylvania, primarily driven by high air pollutants and moderately extreme temperatures. We observed similar patterns in our sensitivity analysis while considering 15 or 25 spatial units to identify potential co‐exposure clusters (Figure S8 in Supporting Information S1).

### 3.5 Bivariate Mapping of Pediatric Vulnerability and Co‐Exposure Anomalies

**Figure 4:**
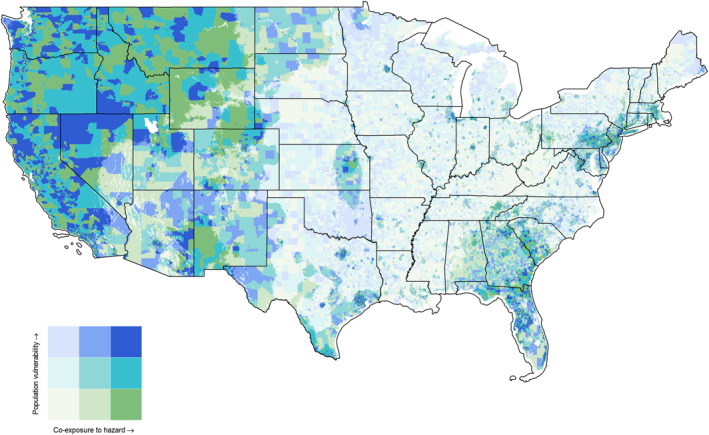
Pediatric risk from potential co‐exposure to ambient air pollutants and extreme temperature. Refer Figure S9 in Supporting Information S1 for high risk areas.

High‐risk areas for pediatric vulnerability and co‐exposure to environmental hazards were observed across multiple U.S. regions, with particularly dense concentrations in portions of the West, South, and Southeast, and in selected urban and rural communities in the Northeast and Midwest (Figure 4; Figure S9 in Supporting Information S1).

In the West, California's highest‐risk locations include urban neighborhoods in the southeastern area of Los Angeles County, rural communities in the Central Valley—specifically Fresno, Kern (Bakersfield), and Merced Counties—and Imperial County along the Mexican border. In Nevada, high‐risk areas are concentrated in the urban region of Clark County (Las Vegas) and Washoe County (Reno and Sparks), as well as in rural areas of Lyon, Elko, and Humboldt Counties. Indigenous lands, including the Fort McDermitt Paiute–Shoshone Tribe and the Duck Valley Indian Reservation, also fall within high‐risk classifications.

In the Northwest, mostly concentrated in Oregon, including Multnomah County (Portland), the rural southeastern counties of Harney and Lake, and the indigenous territories of the Burns Paiute and the Klamath Tribes.

In the Southwest, high‐risk areas in Arizona include Maricopa (Phoenix) and Pima (Tucson) Counties, as well as reservation areas in Apache County occupied by the Navajo Nation, the White Mountain Apache Tribe, and the Hopi Tribe. Additional high‐risk locations include Navajo County and rural areas of Gila County.

In the South, the high‐risk areas are in Texas, Mississippi, and Louisiana. In Texas, these include Harris County (Houston), Dallas and Tarrant (Dallas–Fort Worth area) Counties, border counties such as Cameron (Brownsville), Hidalgo (McAllen), Starr, Webb, and Willacy, as well as Jefferson County (Beaumont–Port Arthur) and Brooks County. In Mississippi, high‐risk counties include Hinds (Jackson), Leflore (Greenwood, rural Delta), Washington, Sunflower, Bolivar, and Humphreys. In Louisiana, the following parishes are identified as high‐risk: Orleans Parish, East Baton Rouge Parish (Baton Rouge), St. John the Baptist Parish, Plaquemines Parish, Tensas Parish, and St. Bernard Parish.

Within the Ohio Valley, high‐risk areas are primarily located in Illinois, notably in Cook (Chicago), St. Clair, Alexander (rural south), Pulaski, and Jackson (Carbondale) Counties.

In the Southeast, Alabama's high‐risk locations include Jefferson (Birmingham), Mobile, Dallas (Selma) Counties, and rural Black Belt counties such as Lowndes, Sumter, Greene, and Wilcox. In Georgia, high‐risk counties include Fulton (Atlanta), DeKalb, Dougherty (Albany, rural south), Early, Clayton, Randolph (rural southwest), and Peach. In Florida, high‐risk areas are located in Miami‐Dade, Broward (urban South Florida), and rural counties, including Gadsden (the panhandle), Glades (southwest Florida), Putnam, and Hendry. High‐risk areas in South Carolina include Florence, Orangeburg, Dillon, Marion, and Williamsburg Counties (rural coastal plain). North Carolina's high‐risk areas include Mecklenburg (Charlotte), Cumberland (Fayetteville), Robeson (rural eastern areas, including the Lumbee indigenous area), Halifax, and Northampton Counties.

In the Northeast, high‐risk areas are in Pennsylvania and New York. In Pennsylvania, these include Philadelphia, Allegheny (Pittsburgh), Cambria, Northumberland (rural central Pennsylvania), and Schuylkill Counties. In New York, high‐risk counties include Kings (Brooklyn, NYC), Bronx, Erie (Buffalo), St. Lawrence (rural north), and Franklin (rural north).

## 4 Discussion

Our study compared five dimensionality reduction methods to develop a pediatric vulnerability index. We observed that latent variable principal component analysis (PCA) explained approximately 23% of the variance in the vulnerability data. Between 2012 and 2024, extreme temperature anomalies were prevalent in the Southwest and Nevada, while rural areas of the Ohio Valley showed cooling patterns. From 2010 to 2020, PM_2_._5_ anomalies were prevalent in the western and northern regions, with most of the CONUS showing stable or improving air quality except for slight increases in some western states. Black carbon anomalies declined nationwide, except in California, Nevada, Arizona, and New Mexico, where slight increasing trends were observed.

Our co‐exposure analysis identified that 36% of the CONUS had statistically significant hotspots for combined exposure to extreme temperature and air pollution, 40% were coldspots, and 24% showed null results. Hotspots were concentrated in the western states (West, Northwest, Southwest), with additional clusters in Colorado, Wyoming, Montana, and eastern Pennsylvania. These areas were characterized by high exposure to both temperature and air pollutants, highlighting regions where children may face disproportionate risks from coexposure to extreme temperatures and air pollutants. High‐risk areas with higher pediatric vulnerability and co‐exposure to environmental hazards were identified in both urban and rural settings. Notable regions included California's Central Valley, southern Nevada, indigenous lands in Nevada and Oregon, urban and indigenous areas in Arizona, major urban centers and rural Delta communities in Texas, Mississippi, and Louisiana, urban Chicago and rural southern Illinois, and counties across Alabama, Georgia, Florida, South Carolina, North Carolina, Pennsylvania, and New York. This mapping underscores that pediatric populations in diverse settings—indigenous, coastal, agricultural, and industrial—face disproportionate risks from combined exposures.

Potential underlying factors for these patterns likely reflect underlying structural and contextual factors that concentrate racial/ethnic minorities in neighborhoods with greater environmental burden (Bailey et al., 2017; Berberian et al., 2022; Hoffman et al., 2020). Historical lending practices by the Home Owners' Loan Corporation (HOLC) drove the low‐income and minority communities closer to air pollutant sources such as the traffic corridors, industrial facilities, and also limited access to green space that may exacerbate environmental exposures (Chakraborty et al., 2023; Mikati et al., 2018; Morello‐Frosch & Jesdale, 2006). These reasons help explain why children living in underserved communities who already face barriers to health care access and home cooling and exposed to elevated environmental factors, resulting in high susceptibility to develop adverse health outcomes.

In agricultural regions such as the Central Valley and the rural Delta, children may also experience combined exposures to pesticides, dust, wildfire smoke, and extreme heat (Bennett et al., 2025; Parenteau et al., 2022; von Ehrenstein et al., 2019). Children living in indigenous lands or industrial communities face additional vulnerabilities: poor infrastructure, limited health services, and inadequate climate adaptation resources often exacerbate these environmental burdens (National Academies of Sciences, Engineering, and Medicine, 2023). Together, these compounding exposures and inequities explain why children in underserved communities—who already experience higher baseline rates of adverse health conditions, limited access to health care, and fewer resources for home cooling—are susceptible to adverse health outcomes.

We observed moderate heterogeneity between our risk maps and the existing environmental health indices. The National Integrated Heat Health Information System‐Heat Vulnerability Index (HVI) and Climate Vulnerability Index (CVI) both identify the Southwest as a region with elevated heat risk, consistent with our findings of extreme temperature anomalies throughout Arizona, Nevada, and New Mexico. However, these indices highlight the Southwest as a uniformly high‐vulnerability region, rather than identifying microregions at high risk. Both the HVI and CVI indices use a static temperature threshold (days above 35°C) at the census tract scale, which may lead to spatial smoothing and the failure to capture acclimatization at the microregional scale (Bradford et al., 2015). Although the CVI includes a range of environmental hazards beyond extreme heat and air pollution, the indicator variable includes heterogeneous environmental hazards, which may introduce noise.

The EJI identified high environmental disparity burden concentrated in the upper Midwest industrial corridor (Illinois, Indiana, Ohio), the South (Texas, Louisiana, Mississippi, Alabama), and scattered regions in Appalachia and the Pacific Northwest, with localized high‐burden areas in Oregon, Oklahoma, and West Virginia. Whereas the COI 3.0 identified high‐risk areas in major metropolitan areas along the Northeast corridor (Boston, New York, Philadelphia, and Washington, DC) and scattered Great Lakes cities (Chicago, Detroit, Minneapolis). Both the EJI and COI are multifaceted, representing a spectrum of environmental factors at a coarse spatial resolution (McKenzie et al., 2024; Noelke et al., 2025). We observed moderate overlap in the EJI with areas similar to our results in the northwest. Furthermore, the overall risk patterns in our study did not overlap with COI 3.0 or with the findings reported by Manware et al. (2022).

The indices CVI, EJI, and COI were developed using a weighted index method that does not provide a metric for comparison with our results. Whereas Manware et al. used PCA to develop the heat index, the authors reported cumulative variance explained rather than the individual principal components. This resulted in limitations in directly comparing the existing indices with our findings. We believe the heterogeneous patterns between our extreme heat patterns compared to Manware et al. could be primarily driven by the exposure metric, where we used NOAA climate normals as a threshold, whereas Manware et al. relied on the calculation of the most frequently occurring temperature as a threshold. Furthermore, our study periods differ from those of Manware et al., using 2017–2020 temperature data and 2015–2019 American Community Survey estimates, compared with our 2012–2024 temperature window and 2021 ACS data; shifts in both climatic trends and population characteristics over this interval may influence the spatial distribution of vulnerability. Additionally, our vulnerability index was focused on the pediatric population characteristics, whereas Manware et al. modeled using overall population characteristics, which may lead to heterogeneous patterns.

This study adds value to the literature by comparing five dimensionality reduction methods to construct the pediatric vulnerability index and by incorporating the IPCC framework to identify neighborhoods at higher risk of coexposure to extreme heat and air pollutants. This approach enabled us to identify high‐risk areas in the southeastern and northeastern coastal regions of the CONUS. In contrast to COI 3.0, we measured the frequency of hot days during the summer season, considering the NOAA's historic Climate Normals (monthly), which account for human adaptive capacity and resilience to extreme temperatures by geography and intra‐seasonal variance. Our analysis can inform the development of decentralized heat action plans (HAPs) in the CONUS, especially given that only ∼60% of HAPs in the US have focused outreach strategies for pediatric populations (Randazza et al., 2023). Additionally, our findings may support resource allocation in areas with sparse coverage of cooling centers, particularly in the Northern Rockies and Plains, the Northwest, Southwest, Southeast, and Northeast (Adams et al., 2023).

Our study has certain limitations. The pediatric vulnerability index showed modest correlations with the original variables included in this study, suggesting a potential loss of information from these variables—a common phenomenon observed when constructing latent variables (Bradford et al., 2015; Conlon et al., 2020; Cutter & Emrich, 2017; Jolliffe & Cadima, 2016). Resampling hazard layers may introduce artificial precision, potentially overstating fine‐scale variability; future validation studies at native‐resolution observations are needed. There is a temporal mismatch between data sets included in this study (vulnerability: 2021 ACS; temperature: 2012–2024; pollutants: 2010–2020), which limits direct alignment, though long‐term averaging mitigates short‐term noise. Additionally, we limited the spatial resolution of the pediatric vulnerability index to the census tract scale due to the availability of variables from the US Census database. Although the results presented in this study are based on long‐term patterns, the temperature and air pollution data overlap for 9 years, which may limit the robustness of direct comparisons across panels. This study did not include indicators of pediatric health burden related to extreme heat/air pollutant exposures; further studies incorporating pediatric morbidity or mortality data from these exposures are needed to validate our findings. Additionally, because our focus is on the meteorological summer months to highlight extreme heat‐related risks, the potential implications of projected seasonal shifts (Wang et al., 2021) and extreme cold‐related risks are understudied in our approach.

## 5 Conclusions

In this study, we developed a pediatric vulnerability index at the US census tract scale by comparing five dimensionality reduction methods. Using the IPCC risk framework, we mapped areas with higher co‐exposure to extreme heat and air pollution and higher pediatric vulnerability. Overall, we observed high‐risk neighborhoods concentrated in the rural or small‐town areas of Oregon, Nevada, Arizona, Utah, New Mexico, Colorado, and Wyoming. These findings complement existing national indices identifying potential high‐risk areas that can guide hypothesis generation, targeted adaptation planning, and future work linking these patterns to pediatric exposure and health outcome data.

## Conflict of Interest

The authors declare no conflicts of interest relevant to this study.

## Supporting information

Supporting Information S1

## Data Availability

Raw data are publicly available from the databases at Durre, Squires, et al. (2022) and Wei, Wang, and Li (2023) and Wei, Wang, Li, et al. (2023). Processed data, model output, and analytic code are available at Puvvula, 2025.
