# Supplementary material for: Pediatric Risk Mapping From Co‐Exposure to Extreme Temperatures and Air Pollutants
Source: Geohealth. 2026 May 30;10(6):e2025GH001743. doi: 10.1029/2025GH001743 (PMC13239795; doi:10.1029/2025GH001743)
Supplement: Supplementary file 1 — Supporting Information S1 [file GH2-10-e2025GH001743-s001.docx]

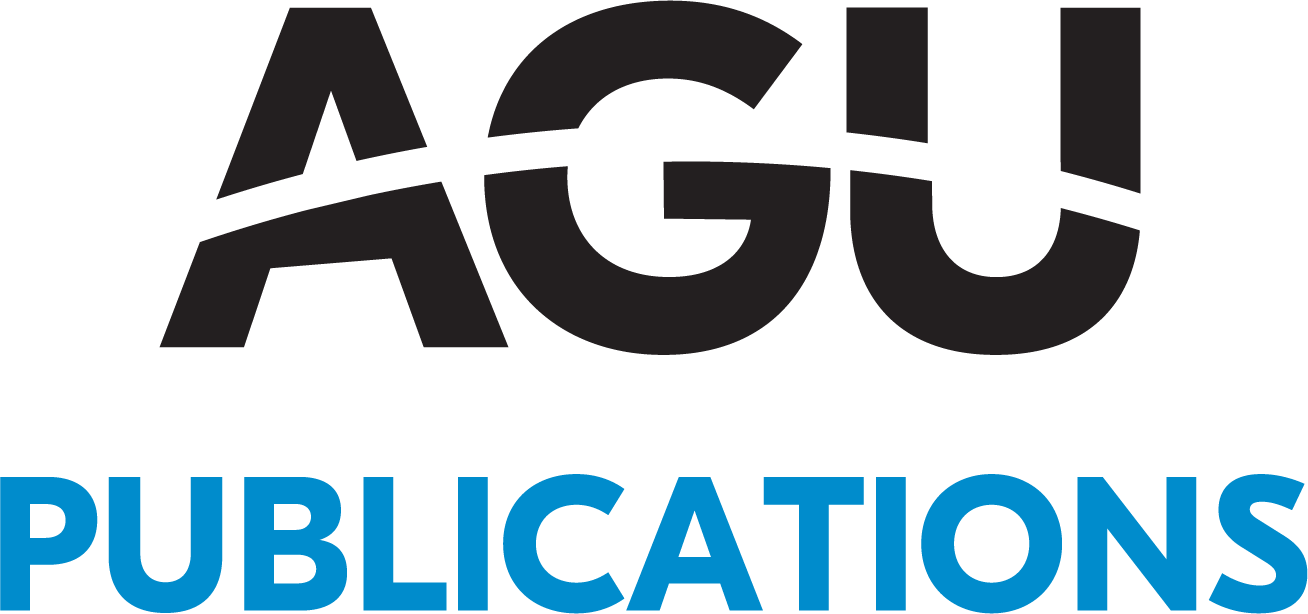


*GeoHealth*

Supporting Information for

**Pediatric Risk Mapping from Co-Exposure to Extreme Temperatures and Air Pollutants**

Jagadeesh Puvvula^1,2^, Jonathan Szeto^3^, Jabeen Taiba^4^, Jennifer Ko^1^, Jesse Bell^4,5,6^, Wei-Ting Hwang^1^, Rebecca Simmons^7,8,9^, Marilyn Howarth^3^, Sameed Khatana^2,10,11^, Kyle Jackson^12^, Aimin Chen^1^

^1^Department of Biostatistics, Epidemiology and Informatics, Perelman School of Medicine, University of Pennsylvania, Philadelphia, PA

^2^The Leonard Davis Institute of Health Economics, University of Pennsylvania, Philadelphia

^3^Perelman School of Medicine, University of Pennsylvania, Philadelphia

^4^Department of Environmental, Agricultural, and Occupational Health, College of Public Health, University of Nebraska Medical Center, Omaha, NE

^5^School of Natural Resources, University of Nebraska-Lincoln, Lincoln, NE

^6^Daugherty Water for Food Global Institute, University of Nebraska, Lincoln, NE

^7^Center for Research on Reproduction and Women’s Health, University of Pennsylvania, Philadelphia, PA

^8^Department of Pediatrics, Perelman School of Medicine at the University of Pennsylvania, Philadelphia, PA

^9^Division of Neonatology, Children’s Hospital of Philadelphia, Philadelphia, PA

^10^Division of Cardiovascular Medicine, Perelman School of Medicine, University of Pennsylvania, Philadelphia

^11^Penn Cardiovascular Outcomes, Quality, and Evaluative Research Center, Perelman School of Medicine, University of Pennsylvania, Philadelphia

^12^ Division of Transplant Surgery, Hospital of the University of Pennsylvania, Philadelphia, PA

Contents of this file: 9 figures and 3 tables (14 pages)

[**Figure S1.** Monthly maximum temperature climate normal – meteorological summer months (JJA). 3](#_Toc220508258)

[**Figure S2.** Pediatric health risk definition. This risk framework was adapted from the IPCC. 4](#_Toc220508259)

[**Figure S3.** Metrics from the PCA model. 5](#_Toc220508260)

[**Figure S4.** Annual temperature anomaly trend over 2012-2024. 6](#_Toc220508261)

[**Figure S5.** Annual PM_2.5_ anomaly trend over 2010-2020. 7](#_Toc220508262)

[**Figure S6.** Western CONUS annual PM_2.5_ anomaly trend over 2010-2020. 8](#_Toc220508263)

[**Figure S7.** Annual black carbon anomaly trend over 2010-2020. 9](#_Toc220508264)

[**Figure S8.** Sensitivity analysis for co-exposure hotspots. 10](#_Toc220508265)

[**Figure S9.** Areas that overlap high environmental hazards and high pediatric vulnerability. 11](#_Toc220508266)

[**Table S1.** Data included in this study 14](#_Toc213747972)

[**Table S2.** PubMed query as of Aug 14, 2025 12](#_Toc213747973)

[Table S3. Summary of published literature 13](#_Toc213747974)

| **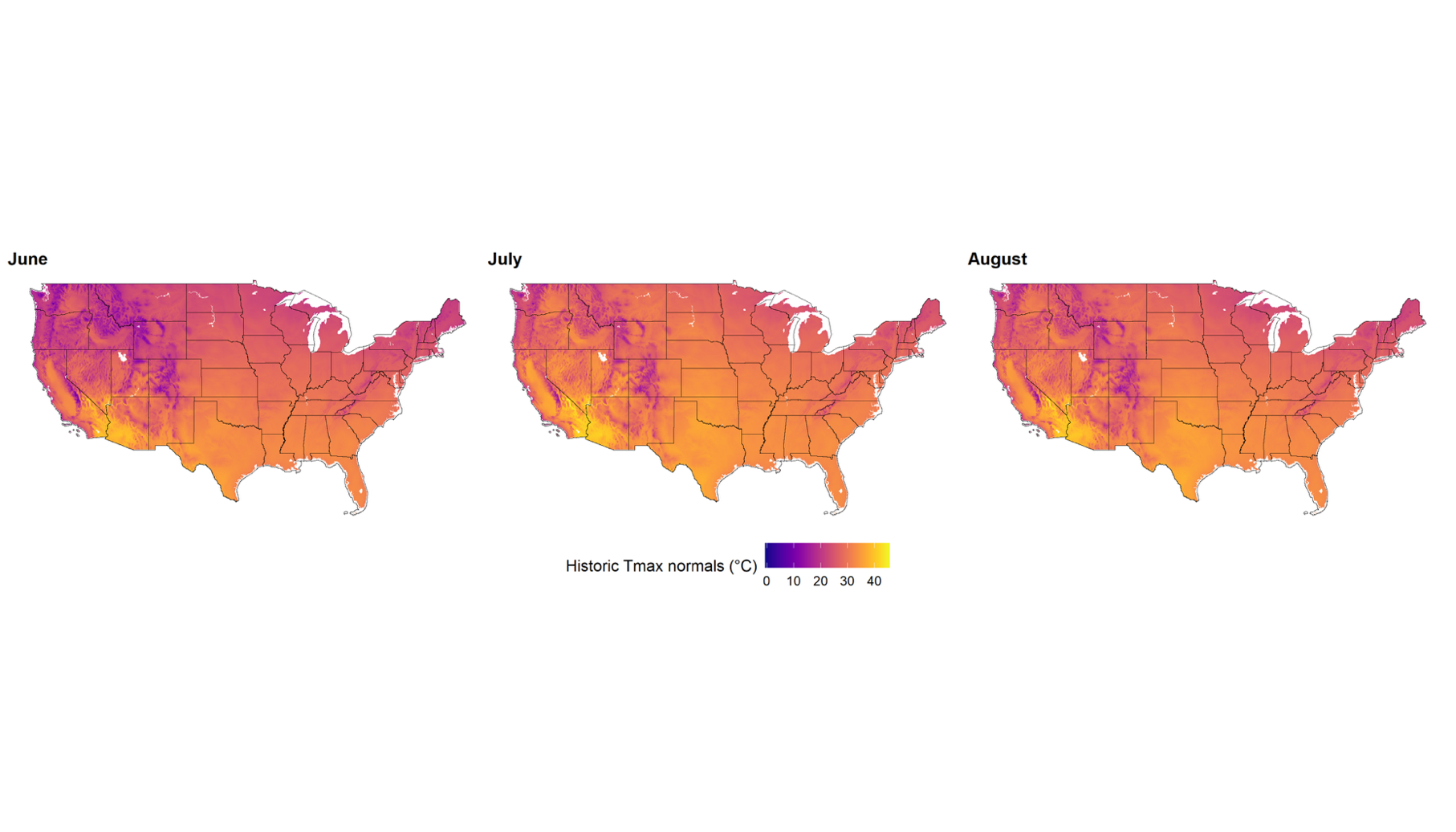** |
| --- |
| Figure S1. Monthly maximum temperature climate normal – meteorological summer months (JJA). |

**Figure S2.** Pediatric health risk definition. This risk framework was adapted from the IPCC. **
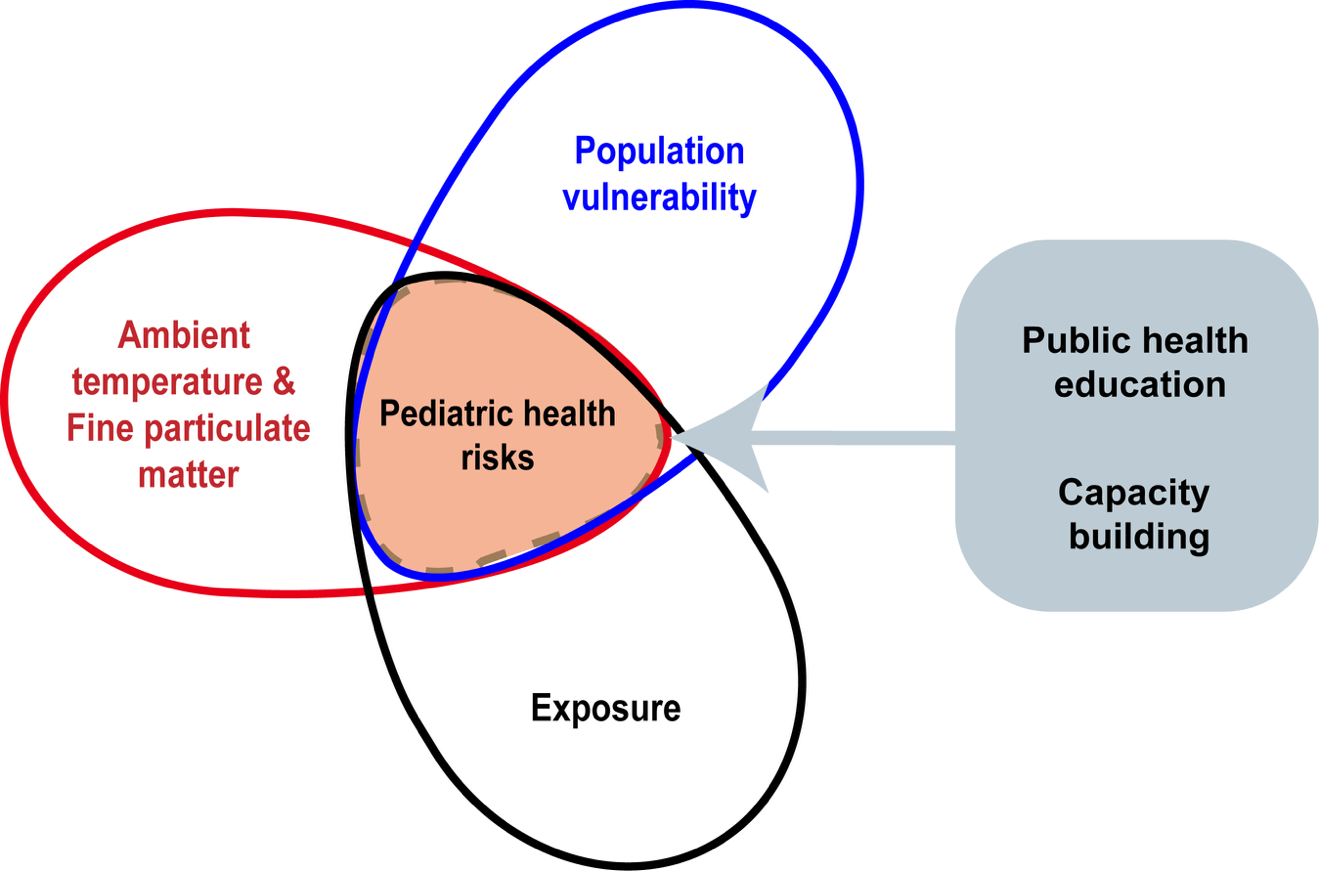
**

| **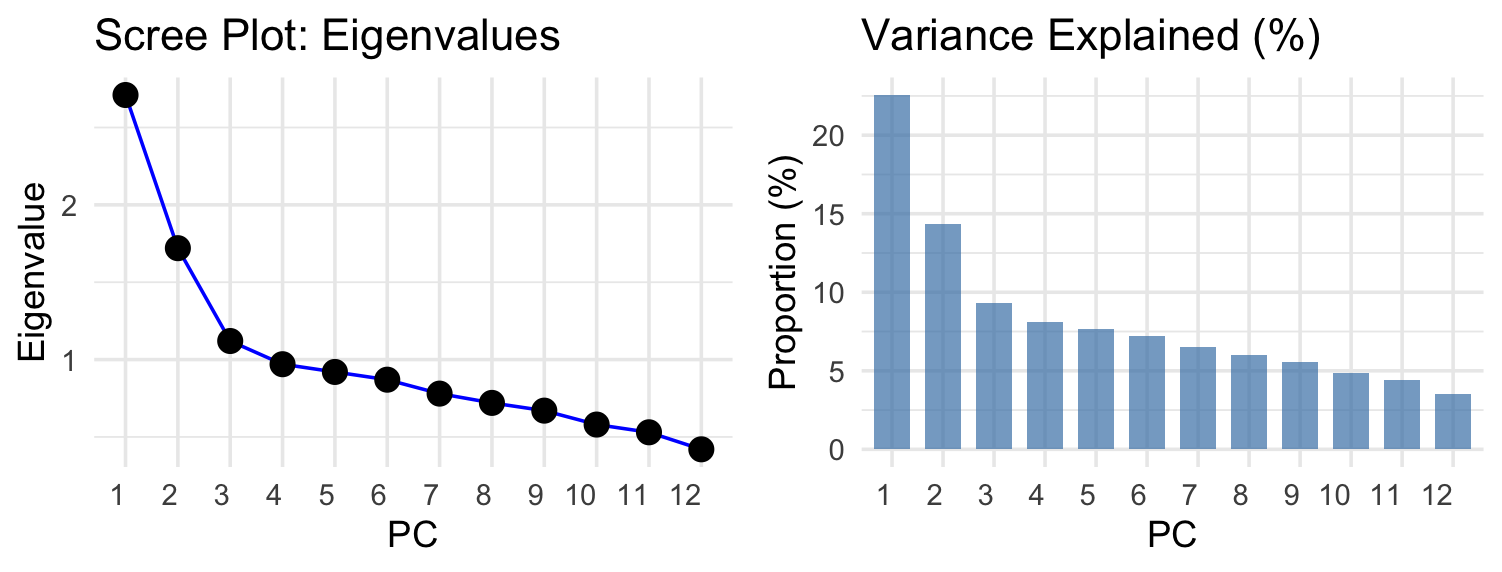** |
| --- |
| **Figure S3.** Metrics from the PCA model. |

| **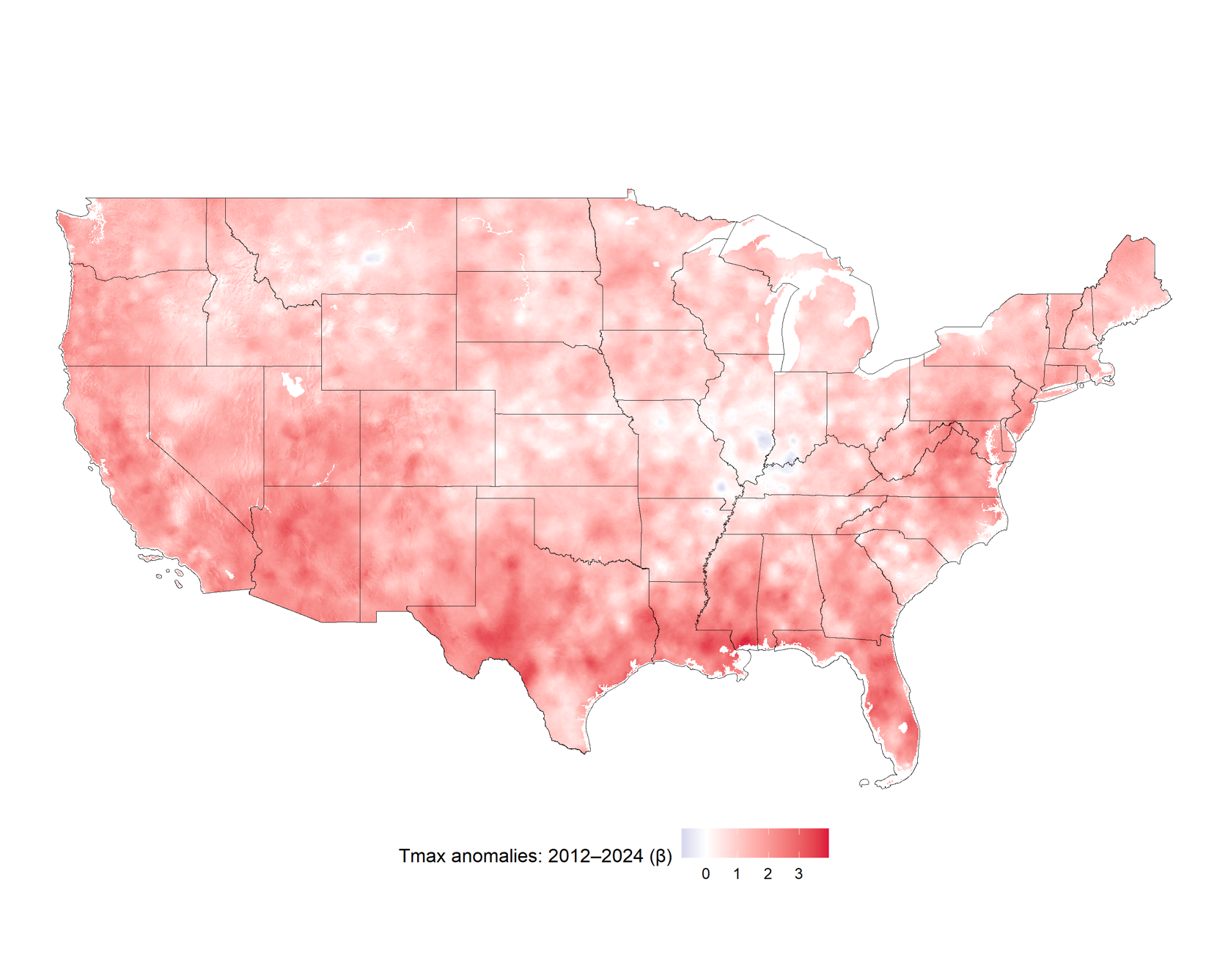** |
| --- |
| **Figure S4.** Annual temperature anomaly trend over 2012-2024.  Trend was calculated using the year as a predictor to estimate the frequency of annual maximum temperature anomalies. The beta estimates are interpreted as every year increase is associated with the frequency of maximum temperature anomalies. Blue shades represent the cooling trend, and red shades represent the warming trend. |

| 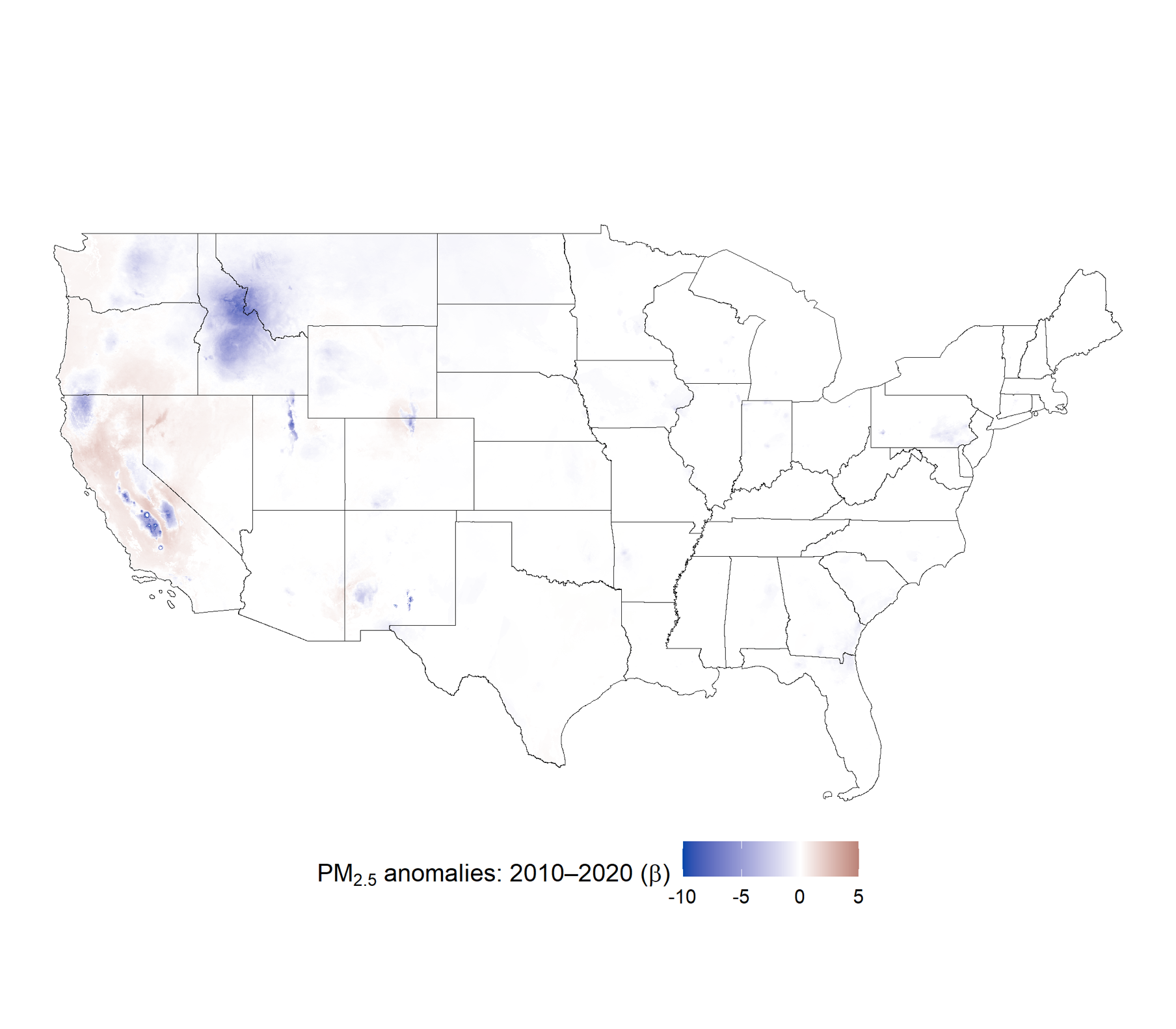 |
| --- |
| **Figure S5.** Annual PM_2.5_ anomaly trend over 2010-2020.  Trend was calculated using the year as a predictor to estimate the frequency of annual PM_2.5_ anomalies. The beta estimates are interpreted as every year increase is associated with the frequency of PM_2.5_ anomalies. Blue shades represent the decline in PM_2.5_, and red shades represent worsened PM_2.5_. Refer to Figure S6 for paned in view of the west. |

| **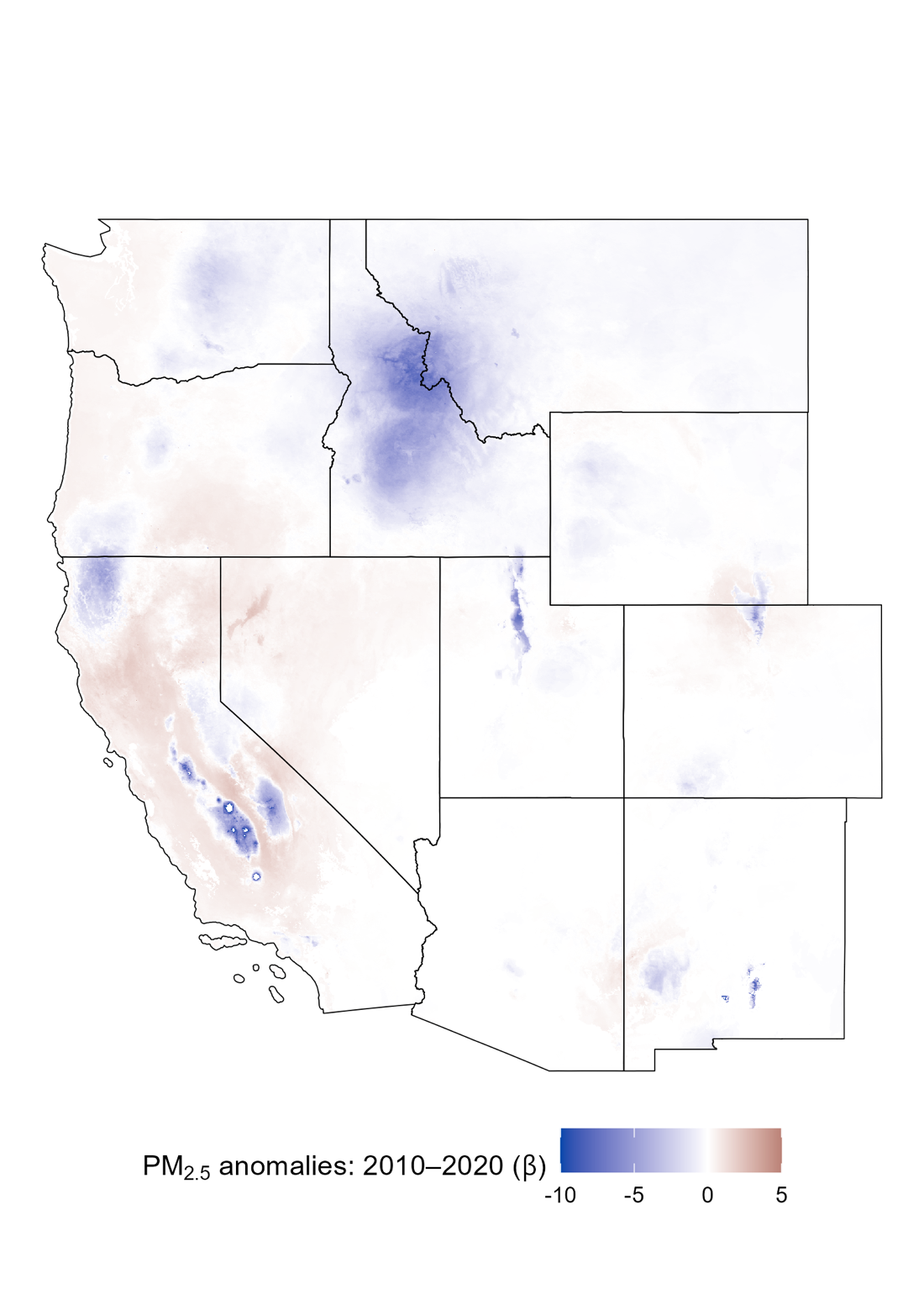** |
| --- |
| **Figure S6.** Western CONUS annual PM_2.5_ anomaly trend over 2010-2020. |

| 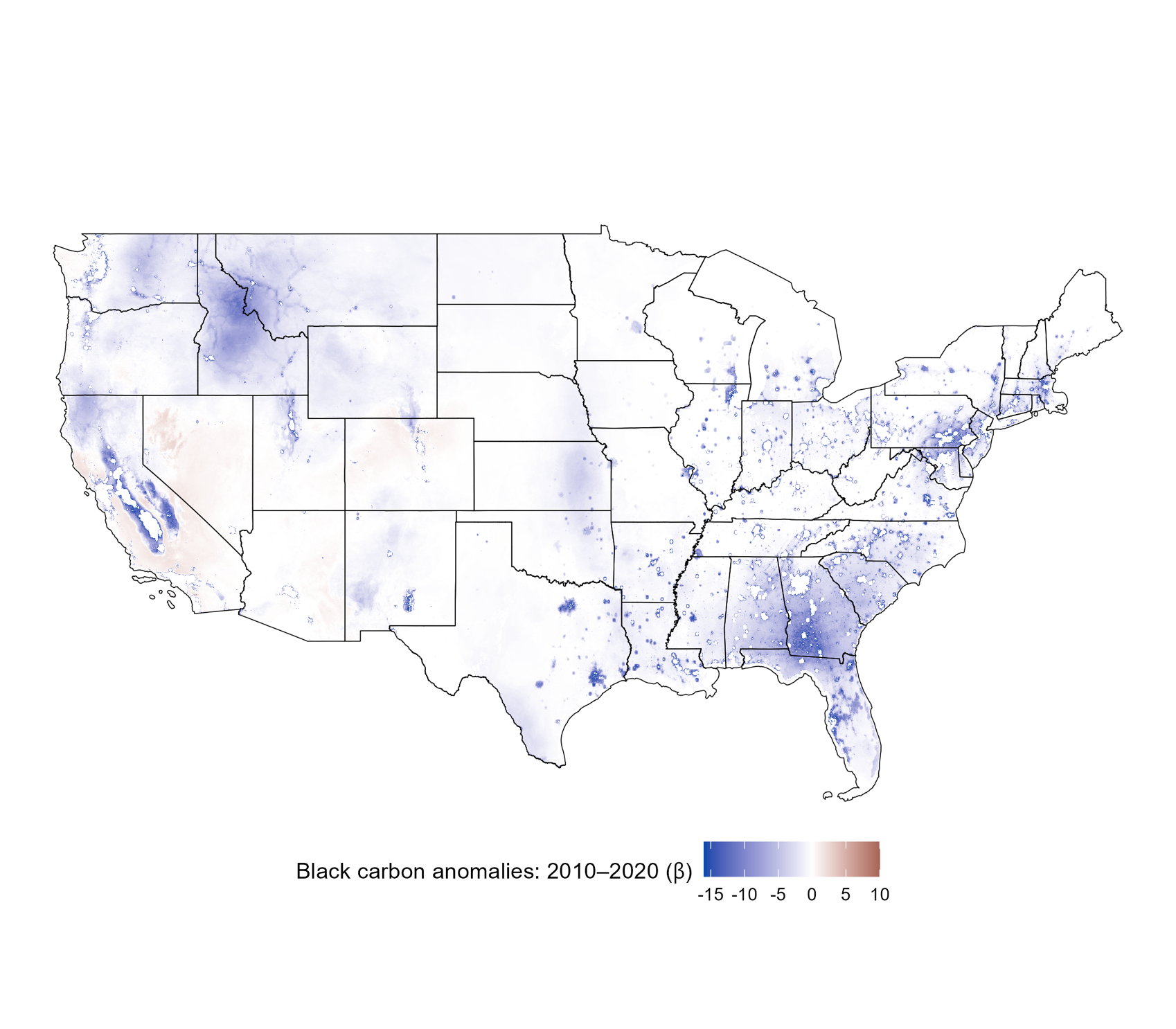 |
| --- |
| **Figure S7.** Annual black carbon anomaly trend over 2010-2020.  The trend was calculated using the year as a predictor to estimate the frequency of annual black carbon anomalies. The beta estimates are interpreted as every year increase is associated with the frequency of black carbon anomalies. Blue shades represent the decline in black carbon, and red shades represent worsened black carbon. |

| **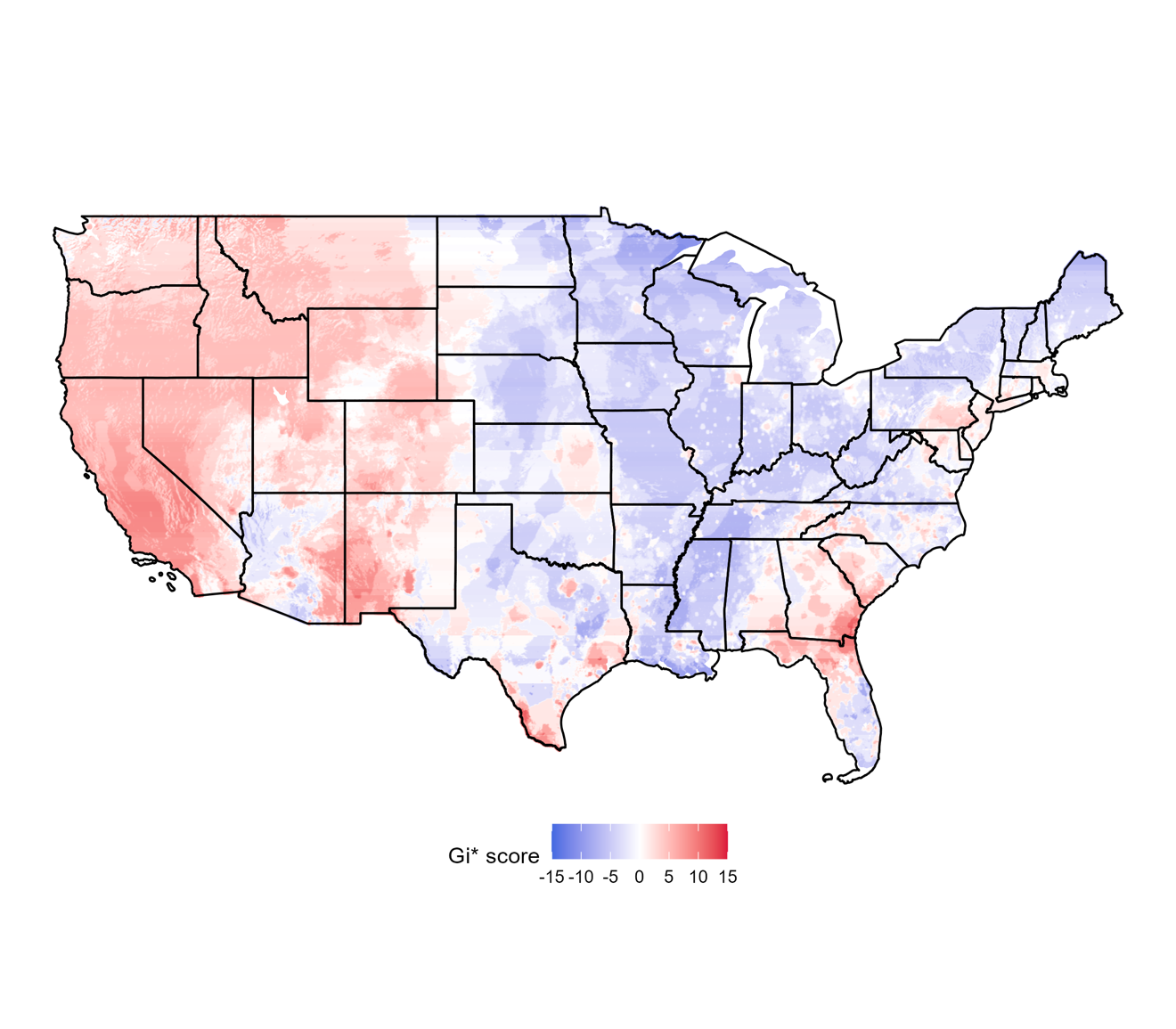**  **A** |
| --- |
| **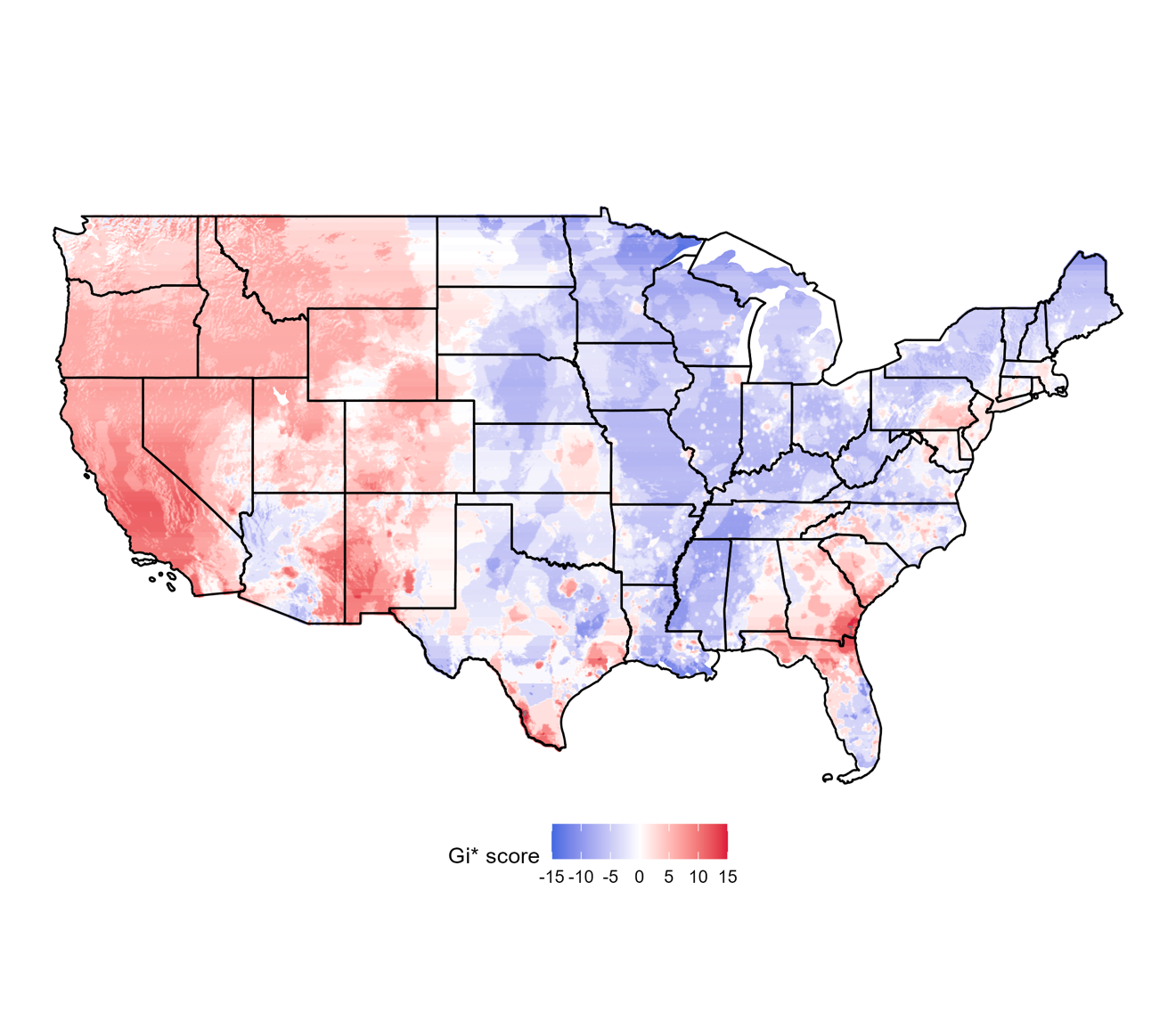**  **B** |
| **Figure S8.** Sensitivity analysis for co-exposure hotspots.  .Panel-A represents analysis using K=15 neighbors and Panel-B represents analysis using K=25. |

| **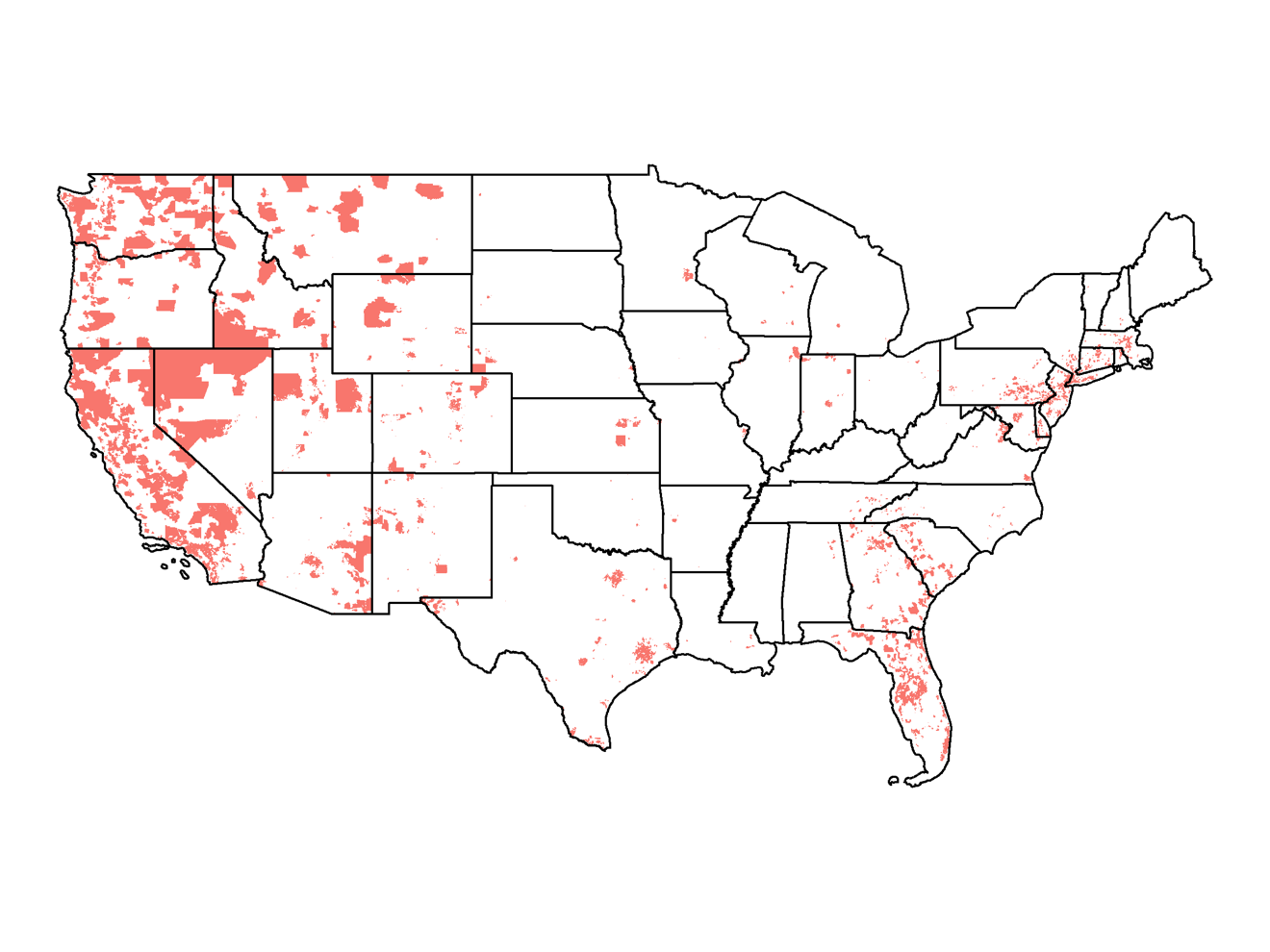** |
| --- |
| **Figure S9.** Areas that overlap high environmental hazards and high pediatric vulnerability. |

| **Table S1.** PubMed query as of Aug 14, 2025 |
| --- |
| ( ("heat"[TIAB] OR "temperature"[TIAB] OR "air pollution"[TIAB] OR "PM2.5"[TIAB] OR "particulate matter"[TIAB] OR "black carbon"[TIAB]) AND ("vulnerability"[TIAB] OR "vulnerable populations"[TIAB] OR "fragility"[TIAB] OR "social vulnerability"[TIAB] OR "heat risk"[TIAB] OR "heat vulnerability index"[TIAB] OR "air vulnerability index"[TIAB] OR "environmental vulnerability index"[TIAB]) AND ("neighborhood"[TIAB] OR "neighborhoods"[TIAB] OR "census tract"[TIAB] OR "census tracts"[TIAB] OR "census blocks"[TIAB] OR "US Census"[TIAB] OR "census data"[TIAB] OR "community characteristics"[TIAB]) ) AND "United States"[MeSH Terms] |
| Retrieved 66 articles |

| Table S2. Summary of published literature | | | | |
| --- | --- | --- | --- | --- |
| Article | Environmental hazard | Geographic area | Geographic resolution | No. of Vulnerability factors |
| Harlan et al., 2006 | Extreme temperature (Central Arizona–Phoenix Long-Term Ecological Research Project ~30 sq m) | Pheonix, AZ | Block group | 5 |
| Reid et al., 2009 | Extreme temperature  (Land cover and indoor characteristics) | USA | Census tracts | 10 |
| Sadd et al., 2011 | Air pollution (Average PM_2.5_ during 2004-2006 –California Air Resources Board) | South California | Census tracts | 10 |
| Johnson et al., 2012 (+) | Extreme temperature (Landsat 5 Thematic Mapper – maximum solar radiance ~30-120m) | Chicago, IL | Census blocks | 16 |
| Aubrecht et al., 2013 (+) | Extreme temperature  (Max temperature > 30℃ - GHCN-D) | DC area | Census blocks | 6 |
| Weber et al., 2015 (+) | Extreme temperature  (MODIS – land surface temperature ~1KM) | Philadelphia, PA | Block group | 11 |
| Binita et al., 2015 (+) | Heat, drought, flood (independently) (Spatial Hazard Events and Losses Database for the United States) | Georgia | Counties | 12 |
| Eisenman et al., 2016 (+) | Extreme temperature (Max temperature anomalies between June–August – NOAA 12KM) | Maricopa County, AZ | Census tracts | 13 |
| Christenson et al., 2017 (+) | Heat and air pollution (independently) (Surface temperature – PRISM ~4km; PM_2.5_ – EPA Air quality monitors) | Wisconsin,  Milwaukee | Block group | 7 |
| Liévanos et al., 2018 | Variety of environmental exposures (includes air pollution) (long-term average of air pollutants – California Air Resources Board) | California | Census tracts | 8 |
| Nayak et al., 2018 | Extreme temperature  (Land cover) | New York | Census tracts | 11 |
| Koman et al., 2019 | Extreme temperature  (projected temperature anomalies – CMIP3) | Michigan | Census tracts | 5 |
| Samuelson et al., 2020 | Extreme temperature  (land cover and indoor characteristics) | Boston, MA  Phoenix, AZ | Census tracts | 8 |
| Jalazedeh-Fard et al., 2021 (+) | Extreme temperature  (Land cover) | Nebraska | Census tracts | 7 |
| Kannoth et al., 2025 (+) | Air pollution  (New York City Community Air Survey) | New York City, NY | Zip codes | 54 |
| Anderson et al., 2025 (+) | Overall environmental factors (including heat and air pollution) (Average temperature May-Sept: Jefferson County Urban Heat Management Study & PM_2.5_ – CMAQ census tract scale) | Louisville Metro area, KY | Census tracts | 9 |
| +Articles manually included | | | | |

| **Table S3.** Data included in this study | | | |
| --- | --- | --- | --- |
| Data layer | Variables | Variable identifier | Variable of interest |
| **Social determinants^a^** | Proportion of children by racial/ethnic minorities | B01001B  B01001I  B01001D  B01001C, B01001E & B02014 | African American |
|  |  |  | Hispanic or Latino |
|  |  |  | Asian |
|  |  |  | American Indian, Alaska Native, Hawaiian, Other pacific islander, and tribal |
|  | Access to care/transportation | B27001  B08201_002 | Children without health insurance |
|  |  |  | Households without access to a vehicle^c^ |
|  | Financial vulnerability | B22002_003  B17001B  B23007 (010 & 013) | Households with children under 18-years and received Food stamps/SNAP |
|  |  |  | Households below 100% federal poverty level in past 12 months^c^ |
|  |  |  | Children (<=18years) living with unemployed parents |
|  | Other | B16008  B06012_013  B05009_013, B05009_031 | English language barrier (5-17 years) |
|  |  |  | Foreign born^c^ |
|  |  |  | Children living with single parent |
| **Historic Climate Normals** | **Climate Normals**  (summer months) | NOAA-NCEI: Historic (1901-2000) Monthly Gridded Climate Normals at 4 KM | Monthly normal of maximum temperature monthly |
| **Ambient temperature^b^** | **Maximum temperature anomalies** (summer season) | GHCNd at 4 KM spatial resolution (daily) | Frequency of days above the NOAA climate normals (2012-2024) |
| **Ambient fine particulate matter^b^** | **PM_2.5_ and black carbon anomalies** | USHighAirPollutants (USHAP) dataset (Wei et al., 2023) – 1 KM spatial resolution (daily) | Frequency of days with mean concentration PM_2.5_ > 35 μg/m³ and black carbon > 1 μg/m³ |
| ^a^Variables obtained using the 5-year American Community Survey at census tract scale released by the US Census Bureau in 2021. ^b^Remote sensing observations accessed through google earth engine and NOAA databases. ^c^Variables representing overall population. Most social determinant variables were identified from the 4^th^ and 5^th^ National Climate Assessment reports and Ahdoot et al., 2024. | | | |
